# Investigation of the underlying genes and mechanism of familial hypercholesterolemia through bioinformatics analysis

**DOI:** 10.1186/s12872-020-01701-z

**Published:** 2020-09-16

**Authors:** Dinghui Wang, Bin Liu, Tianhua Xiong, Wenlong Yu, Qiang She

**Affiliations:** 1 Department of Cardiovascular, The Second Affiliated Hospital of Chongqing Medical University, No.1 Medical College Road, Shiyou Road Street, Yuzhong District, Chongqing, 400010 People’s Republic of China; 2 Department of Cardiovascular, The Second Affiliated Hospital, Chongqing Medical University, 76 Linjiang Road, Chongqing, 400010 P.R. China

**Keywords:** Familial hypercholesterolemia, LDL-C, Atherosclerosis, Bioinformatics analysis, ITGAL, TLN1

## Abstract

**Background:**

Familial hypercholesterolemia (FH) is one of the commonest inherited metabolic disorders. Abnormally high level of low-density lipoprotein cholesterol (LDL-C) in blood leads to premature atherosclerosis onset and a high risk of cardiovascular disease (CVD). However, the specific mechanisms of the progression process are still unclear. Our study aimed to investigate the potential differently expressed genes (DEGs) and mechanism of FH using various bioinformatic tools.

**Methods:**

GSE13985 and GSE6054 were downloaded from the Gene Expression Omnibus (GEO) database for bioinformatic analysis in this study. First, limma package of R was used to identify DEGs between blood samples of patients with FH and those from healthy individuals. Then, the functional annotation of DEGs was carried out by Kyoto Encyclopedia of Genes and Genomes (KEGG) enrichment analysis and Gene Ontology (GO) analysis. Based on Search Tool for the Retrieval of Interacting Genes (STRING) tool, we constructed the Protein-Protein Interactions (PPIs) network among DEGs and mined the core genes as well.

**Results:**

A total of 102 communal DEGs (49 up-regulated and 53 down-regulated) are identified in FH samples compared with control samples. The functional changes of DEGs are mainly associated with the focal adhere and glucagon signaling pathway. Ten genes (ITGAL, TLN1, POLR2A, CD69, GZMA, VASP, HNRNPUL1, SF1, SRRM2, ITGAV) were identified as core genes. Bioinformatic analysis showed that the core genes are mainly enriched in numerous processes related to cell adhesion, integrin-mediated signaling pathway and cell-matrix adhesion. In the transcription factor (TF) target regulating network, 219 nodes were detected, including 214 DEGs and 5 TFs (SP1, EGR3, CREB, SEF1, HOX13). In conclusion, the DEGs and hub genes identified in this study may help us understand the potential etiology of the occurrence and development of AS.

**Conclusion:**

Up-regulated ITGAL, TLN1, POLR2A, VASP, HNRNPUL1, SF1, SRRM2, and down-regulated CD69, GZMA and ITGAV performed important promotional effects for the formation of atherosclerotic plaques those suffering from FH. Moreover, SP1, EGR3, CREB, SEF1 and HOX13 were the potential transcription factors for DEGs and could serve as underlying targets for AS rupture prevention. These findings provide a theoretical basis for us to understand the potential etiology of the occurrence and development of AS in FH patients and we may be able to find potential diagnostic and therapeutic targets.

## Background

Familial hypercholesterolemia (FH) is one of the commonest inherited metabolic disorders with limited therapies characterized by an abnormally high level of low-density lipoprotein cholesterol (LDL-C) in blood that has been definitely associated with a premature atherosclerosis onset and a high risk of cardiovascular disease (CVD) [1, 2]. Historically, the incidence of heterozygous FH was about 1 in 500 persons [3]. It’s reported that this number may be as high as 1 in 100 in some European and several South African populations [4]. There are about 3.8 million potential FH patients in China, whereas the clinical and genetic data of FH are limited [5, 6]. Studies show that heterozygous FH has the uppermost prevalence of genetic flows that cause prominent premature mortality. Goldstein and Brown in their early work first recognized the genetic basis of the disorder, impaired functioning of the low-density lipoprotein (LDL) receptor [7]. Researches of LDL receptor function have revealed additional mechanisms for the pathogenesis of FH (defects in apoli-poprotein [apo] B impairing binding with the LDL receptor and gain-of-function mutations in proprotein convertase subtulisin/kexin type 9 [PCSK9] that enhance LDL receptor degradation) [8]. In addition, there were many different types of LDLR mutation ascertained in sufferers with FH globally. For instance, large gene mutations and rearrangements took place in the promoter region that impact gene transcription [9]. Nevertheless, the molecular mechanism of atherosclerosis in patients with FH is not completely understood, and FH still acts as a proven vital risk factor for the development of atherosclerosis even coronary heart disease. For the therapy, first-line treatment for patients with heFH is with statins which can decline risk of CHD in heFH by up to about 80% while started as a preventive treatment in early age [10]. However, the long-term safety of statins starting at young age in the pediatric population remains unknown because of the non-functional low-density lipoprotein receptor [1]. Linda Omer et al. indicated that CRISPR/Cas9 mediated gene-editing was likely to be a cutting-edge technology to amend gene mutations attributed to diseases, sequentially ameliorating the symptoms of the sick at risk for CVD [11]. Whereas there is still a substantial residual cardiovascular and inflammatory danger of developing CVD that persists after treatment, especially in patients with FH. These realities have pushed forward the search for new therapies against FH, including novel pharmaceutical drugs or genetic engineering technologies.

In the past few decades, technology of the gene chip research and bioinformatic analysis have been wildly applied to screen genetic alterations on genomic level [12–14]. As is well known, bioinformatics mainly focuses on genomics and proteomics. It analyzes the biological information on structural function in the nucleic acid and protein sequence and seeks out genes and proteins related to diseases [15, 16]. At present, increasing researchers utilized bioinformatics to find the potential molecular mechanisms of diseases related to the targeted treatment. In this study, microarray datasets GSE13985 and GSE6054 were obtained from Gene Expression Omnibus (GEO) and analyzed to obtain differently expressed genes (DEGs) between FH patients and controls. The sample data was re-analyzed using various bioinformatic methods such as DEGs screening, functional enrichment analysis and protein-protein interaction network analysis. We hope to identify the potential markers in FH patients, and explore specific targets that could prevent the progression of atherosclerosis.

## Methods

### Collection of raw data

Gene Expression Omnibus (GEO) comprised various species’ microarrays, gene expression data, and chips, is an open-source, high-throughput genomic database [17]. Two expression profile data sets GSE13985 and GSE6054 in our study were obtained from the GEO database. The RNA expression profiles were both assayed on GPL570 platform, [HG-U133_Plus_2] Affymetrix Human Genome U133 Plus 2.0 Array. GSE13985 data set includes 5 blood samples from patients diagnosed with Familial hypercholesterolemia and five from age, sex, BMI and smoking status matched controls. The GSE6054 date set contains 10 FH monocytes samples and 13 control participants. We converted all probe numbers to gene symbols on the base of the annotation information in the platform. As those data were acquired from a public database, no further approval from the local ethics committee was required.

### Data preprocessing

In order to analyze and process the chip data more conveniently, the primary data were preprocessed using affy package in R language. Next we matched gene probe identification to the corresponding gene symbol. Series matrix files were extracted to assess mRNA expression, and mRNA-seq datasets preprocessed by quantile normalization or log2 transformation. In addition, probes were annotated employing the annotation profile from the platform, and unmatched probes were waived. While multiple probes matched to one gene symbol, the probes’ average values were calculated to be the genetic final expression [18].

### Screening genes of differential expression

DEGs between FH patients and those of matched controls of the two expression profile data sets were screened out respectively using the Linear Models for Microarray (LIMMA; Version: 3.30.3) affy in R package12 [19]. *P* value < 0.05 and (|log2FC| > = 0.5) were defined as threshold values in gene sets GSE13985 and *P* value < 0.05, |log2 (FC)| > =1 in GSE6054. Subsequently we select the common up-regulated and down-regulated DEGs from two datasets.

### Functional and pathway enrichment analysis of DEGs

In order to research the biological functions and pathways of these identified DEGs, we performed the GO term and KEGG pathway enrichment analyses of DEGs using the online tool of The Database for Annotation, Visualization, and integrated discovery (DAVID (https://david.ncifcrf.gov/home.jsp version: 6.8)) [20]. DAVID provides a comprehensive assortment of functional annotation system for explorers to screen biological meanings behind numerous genes. By making use of DAVID, and the categories including biological process (BP), cellular component (CC), molecular function (MF) and KEGG pathways were selected for further analysis.

### Integration of the PPI network and hub gene analysis

Using the Search tool for the retrieval of interacting genes/proteins (STRING) (https://string-db.org/) online database, PPIs network among DEGs were constructed with the threshold of medium confidence > = 0.3. Utilizing topological principles, Molecular complex detection (MCODE) (version 1.5.1), a plug-in for Cytoscape, could mine tightly coupled regions from PPIs. Cytoscape software draws the PPI network. Then MCODE identifies the most important modules in the PPI network graph. The score of each module was calculated using the MCODE algorithm [21]. The criteria for MCODE analysis are as follows: node score cutoff = 0.2, degree cutoff = 2, max depth = 100, MCODE score > 5, and k-score = 2.

### Identification of TF targets

Transcription factor networks were constructed employing the differentially expressed data which reference to the collected validated data via several databases [22]. TF targets were extracted from TRANSFAC database. The regulatory interactions between TF and genes were obtained via Python script. Based on DAVID, regulatory relationships between TFs and targeted-DEGs were predicted using Enrichr, and the TF-target regulatory networks were visualized by Cytoscape.

## Results

### Identification of DEGs

**Fig. 1:**
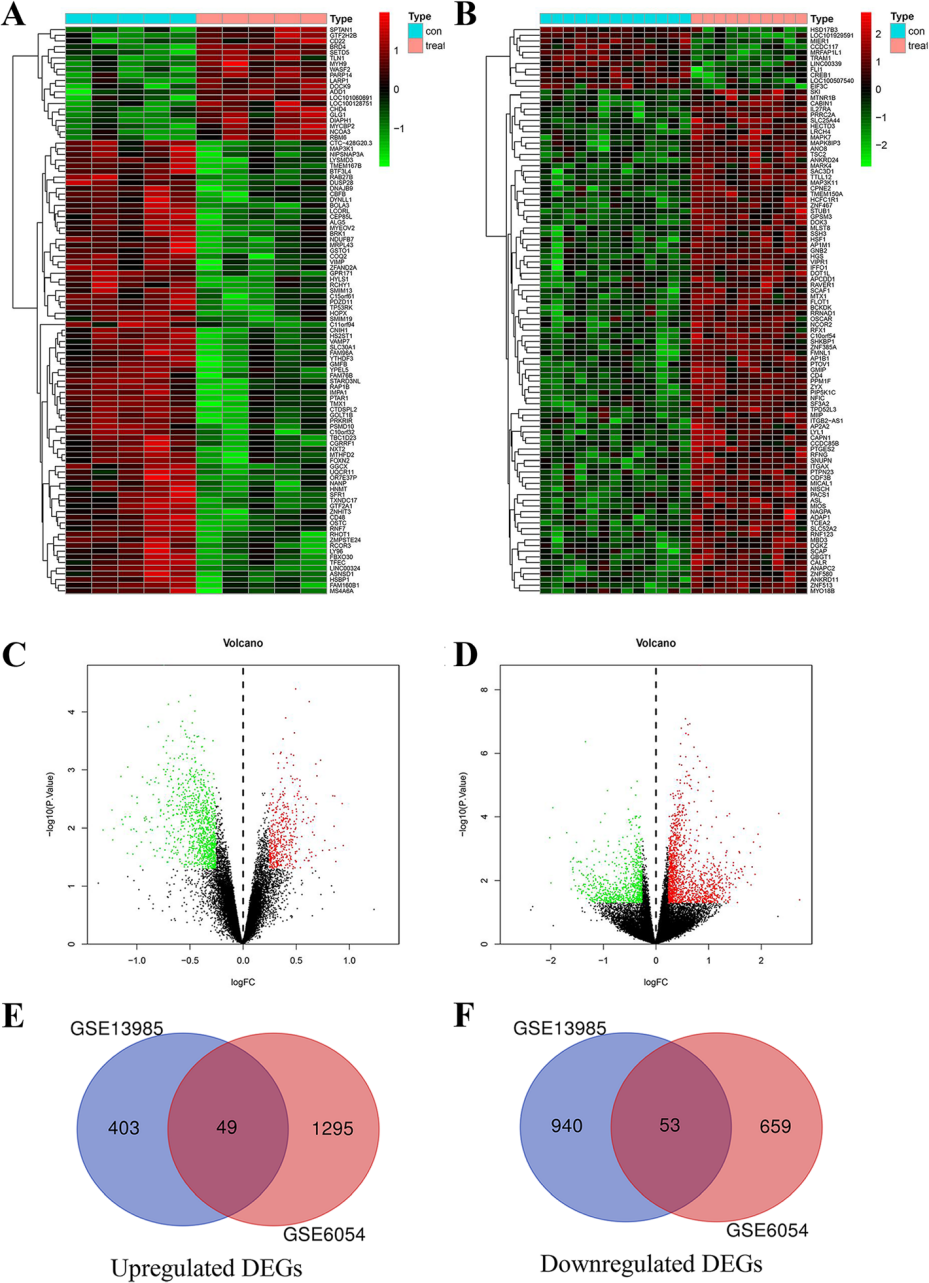
**a** Clustered heat map of DEGs between FH and control samples in GSE13985. **b** Clustered heat map of DEGs between FH and control samples in GSE6054. The abscissa represents different samples, and the ordinate represents different genes. The red boxes indicate up-regulated genes, and the green boxes indicate down-regulated genes. **c** The volcano plot shows the DEGs between FH and control samples in GSE13985. **d** The volcano plot shows the DEGs between FH and control samples in GSE6054. **e** The Venn diagram presents that there are a total of 49 upregulated genes that are simultaneously included in the 2 datasets. **f** The Venn diagram presents that there are a total of 53 downregulated genes that are simultaneously included in the 2 datasets. DEGs: Differentially Expressed Genes

One thousand four hundred forty-five DEGs were identified from dataset GSE13985 comparing FH group to control group (Fig. 1a). Among these, 452 DEGs were up-regulated and 993 DEGs were down-regulated (adjust *p* value < 0.05 and |log2FC| > =0.25). Simultaneously, we identified 2056 DEGs containing 1344 up-regulated DEGs and 712 down-regulated DEGs from GSE6054 (adjust *p* value < 0.05 and |log2FC| > =1) (Fig. 1b). Then, we screen the mutual up-regulated and down-regulated DEGs between GSE13985 and GSE6054, the VENN plot of the results displayed that there were 49 DEGs up-regulated and 53 DEGs down-regulated in both data sets (Fig. 1c,d).

### Functional annotation of DEGs through GO and KEGG analysis

**Fig. 2:**
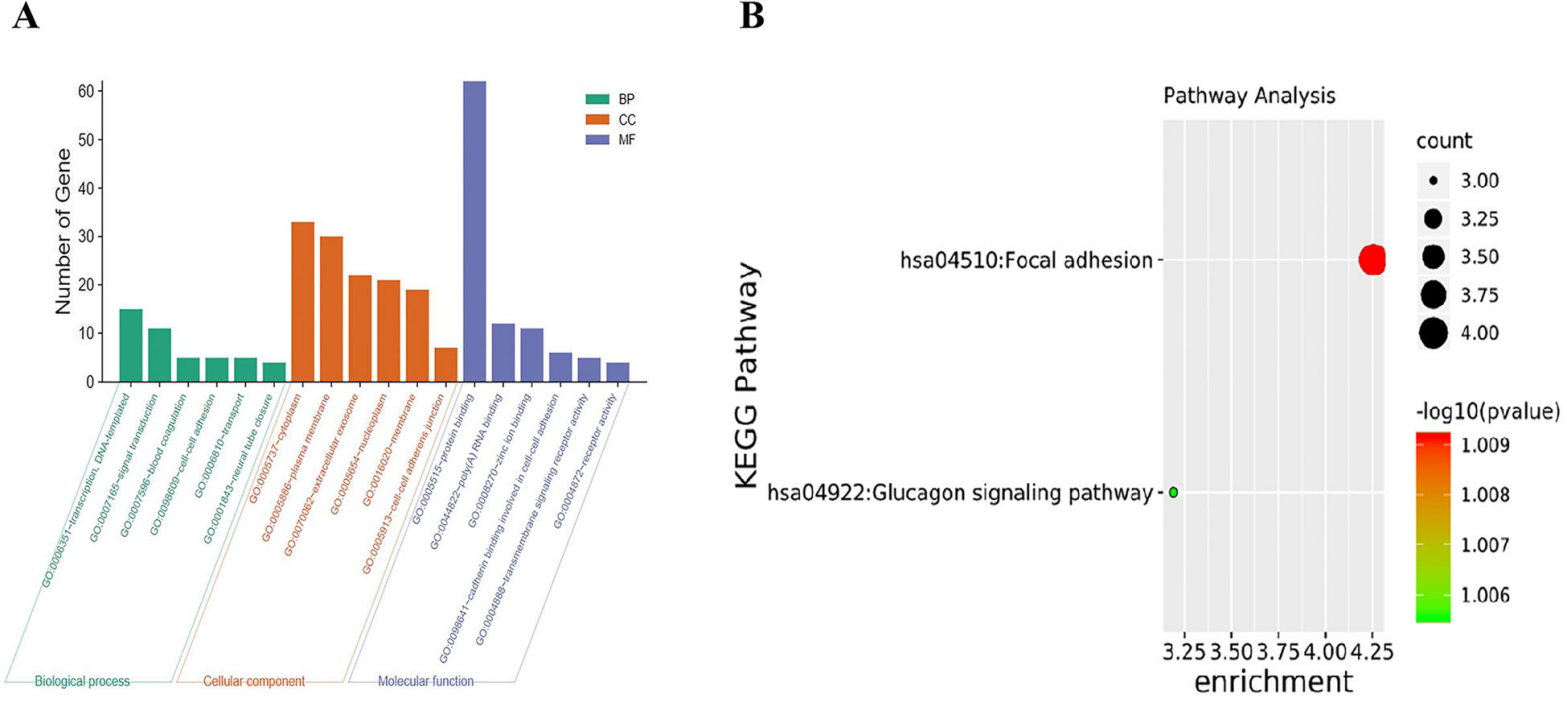
GO terms and KEGG pathway enrichment. **a** GO enrichment analyses of the top six DEGs. **b** The KEGG pathway analysis of DEGs. GO, gene ontology; KEGG, Kyoto Encyclopedia of Genes and Genomes

To uncover the biological classification of DEGs, GO functional and KEGG pathway analyses were performed based on DAVID. GO analysis results demonstrated that the six most enriched biological process annotations were blood coagulation, cell-cell adhesion, ER to Golgi vesicle-mediated transport, integrin-mediated signaling pathway and neural tube closure. Changes obviously enriched in cell component (CC) of DEGs were mainly enriched in cytoplasm, plasma membrane, extracellular exosome, nucleoplasm and membrane. Changes in molecular function (MF) were significantly enriched in protein binding, poly(A) RNA binding, cadherin binding involved in cell-cell adhesion and transmembrane signaling receptor activity (Fig. 2a). The KEGG pathway analysis showed the DEGs were enriched in pathways associated with focal adhere and glucagon signaling pathway (Fig. 2b). More detailed results of GO and KEGG analyses are provided in Table 1.

**Table 1:** The functional enrichment analyses of DEGs ranked by *P*-value

Term	Count	Genes	*P*-Value
GO-BPs			
GO:0001843 ~ neural tube closure	4	TMED2, SKI, PRKACB, VASP	0.006332314
GO:0010942 ~ positive regulation of cell death	3	AKR1C3, UCP2, TRIM13	0.008780361
GO:0007596 ~ blood coagulation	5	P2RX5, F5, ENPP4, PRKACB, WAS	0.012574205
GO:0007229 ~ integrin-mediated signaling pathway	4	ITGAL, ITGAV, PRAM1, ZYX	0.012578224
GO:0038027 ~ apolipoprotein A-I-mediated signaling pathway	2	ABCA7, ITGAV	0.024181939
GO:0051289 ~ protein homotetramerization	3	CRTC2, VASP, GOLGA2	0.034735707
GO:0048208 ~ COPII vesicle coating	3	F5, TMED2, GOLGA2	0.035801513
GO:0035019 ~ somatic stem cell population maintenance	3	BCL9L, SKI, POLR2A	0.040189117
GO:0006888 ~ ER to Golgi vesicle-mediated transport	4	F5, TMED2, GOLGA2, SPTAN1	0.043542377
GO:0098609 ~ cell-cell adhesion	5	PKM, BAG3, VASP, GOLGA2, SPTAN1	0.043641603
GO-CCs			
GO:0005913 ~ cell-cell adherens junction	7	PKM, TLN1, BAG3, ZYX, VASP, GOLGA2, SPTAN1	0.004005355
GO:0032587 ~ ruffle membrane	4	ABCA7, TLN1, ITGAV, IFIT5	0.00665283
GO:0005886 ~ plasma membrane	30	ABCA7, ITGAL, TLN1, LRRC8B, ENPP4, MMP25, PKM, DYSF, ITGAV, BAG3, ANKRD11, CD4, PRKACB, KLRF1, ZYX, FLVCR1, FMNL1, KLRB1, RHBDF2, CD160, EDA2R, VASP, P2RX5, LSP1, PRKD2, NEDD1, F5, IFIT5, P2RY14, CYBRD1	0.010121246
GO:0016020 ~ membrane	19	FMNL1, ITGAL, MOB4, ENPP4, MAP 4 K1, RPS9, PRRC2A, CAD, MMP25, TAPBP, LSP1, PIGK, F5, PEX2, ITGAV, CD4, KLRF1, NCOR2, SPTAN1	0.011799738
GO:0070062 ~ extracellular exosome	22	FMNL1, ITGAL, CRTC2, TLN1, ENPP4, RPS9, MINK1, PRRC2A, CAD, WAS, VASP, AKR1C3, PKM, LSP1, DYSF, CLIC3, UBA1, ITGAV, BEX5, CYBRD1, PRKACB, SPTAN1	0.017083511
GO:1903561 ~ extracellular vesicle	3	PKM, F5, SPTAN1	0.022786951
GO:0017053 ~ transcriptional repressor complex	3	MIER1, SKI, NCOR2	0.027213685
GO:0005654 ~ nucleoplasm	21	CRTC2, SF1, RPS9, CAD, SKI, BOD1L1, POLR2A, FEM1C, PRKD2, NEDD1, HNRNPUL1, MIER1, SRRM2, FYTTD1, ANKRD11, SP4, BCL9L, KPNA5, PRKACB, NCOR2, CHD3	0.02939589
GO:0033116 ~ endoplasmic reticulum-Golgi intermediate compartment membrane	3	F5, TMED2, GOLGA2	0.035966686
GO:0005737 ~ cytoplasm	33	CRTC2, TLN1, MOB4, LRRC8B, MAP 4 K1, TRIM13, PRRC2A, AKR1C3, DNAJC17, PKM, MIER1, BAG3, ANKRD11, HECTD3, ZYX, MTMR6, CHD3, C15ORF39, RPS9, MINK1, SKI, VASP, P2RX5, FEM1C, PRKD2, CLIC3, UBA1, RFK, SP4, BEX5, CMIP, KPNA5, FPGT	0.045491399
GO:0032580 ~ Golgi cisterna membrane	3	MOB4, TMED2, GOLGA2	0.049065899
GO-MFs			
GO:0005515 ~ protein binding	62	RARRES3, TLN1, LRRC8B, HMGN4, TAPBP, PKM, PIGK, DYSF, PEX2, MIER1, BAG3, ZYX, PRKACB, MATK, FLVCR1, GOLGA2, FMNL1, GZMA, SF1, CD160, MINK1, LENG8, WAS, VASP, PRKD2, F5, CLIC3, PRAM1, CYBRD1, KPNA5, CMIP, ITGAL, CRTC2, MOB4, TRIM13, MAP 4 K1, PRRC2A, CIC, POLR2A, TMED2, CD69, ITGAV, HECTD3, LIMD2, CD4, MTMR6, TRAM1, CHD3, EDA2R, RPS9, ZNF26, SKI, FEM1C, MRFAP1L1, HNRNPUL1, UCP2, UBA1, FYTTD1, SP4, BEX5, NCOR2, SPTAN1	0.0000906709830702394
GO:0098641 ~ cadherin binding involved in cell-cell adhesion	6	PKM, TLN1, BAG3, VASP, GOLGA2, SPTAN1	0.014824041
GO:0004888 ~ transmembrane signaling receptor activity	5	KLRB1, P2RX5, CD69, CD4, KLRF1	0.02204474
GO:0044822 ~ poly(A) RNA binding	12	PKM, HNRNPUL1, UBA1, FYTTD1, SRRM2, IFIT5, SF1, RPS9, PRRC2A, ZYX, CHD3, POLR2A	0.023879125
GO:0032393 ~ MHC class I receptor activity	2	CD160, KLRF1	0.02949141
GO:0005522 ~ profilin binding	2	FMNL1, VASP	0.043913166

### PPI network and hub genes analysis

**Fig. 3:**
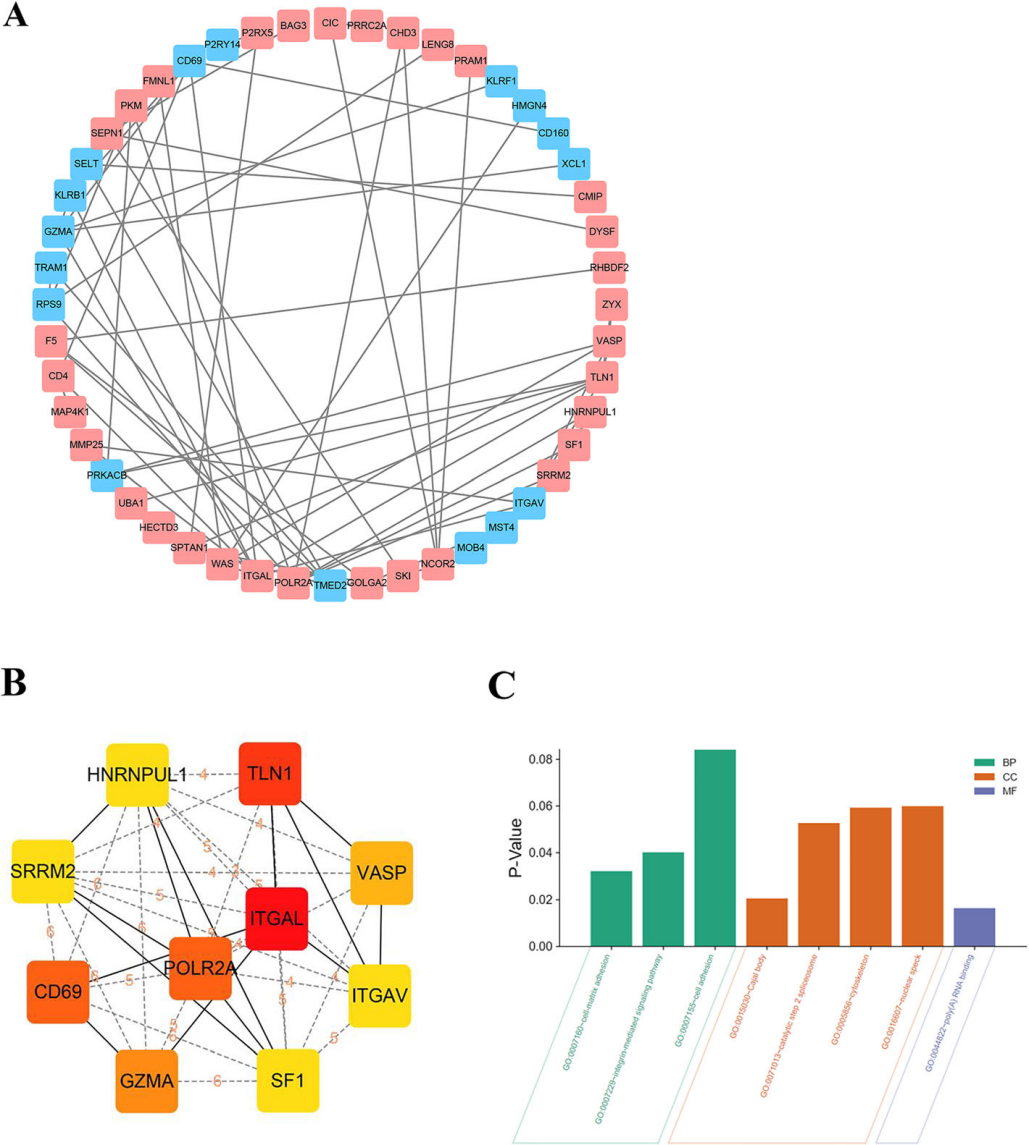
**a** PPI network of signifcantly diferentially expressed genes. Up-regulated genes are marked with light red; down-regulated genes are marked with light green. **b** The top 10 hub genes selected from PPI network. **c** GO enrichment analyses of the hub genes. PPI, protein-protein interaction

Construction of the Protein-protein interactions networks among the DEGs and identification of the most significant modules were performed using the online tool STRING with a cutoff score of ≥0.3 and adjusted through Cytoscape. In total, 65 edges and 48 nodes were involved in our PPI network (Fig. 3a). Utilizing the cytoHubba plugin Cytoscape, a total of 10 genes (ITGAL, TLN1, POLR2A, CD69, GZMA, VASP, HNRNPUL1, SF1, SRRM2, ITGAV) were identified as hub genes with degrees≥6 (Fig. 3b). The names, abbreviations and functions for these hub genes are shown in Table 2. The most significantly enriched BPs containing cell adhesion, integrin-mediated signaling pathway and cell-matrix adhesion. The changes of CCs showed that DEGs were mainly enriched in the nuclear speck, cytoskeleton, catalytic step 2 spliceosome and Cajal body. The changes of MFs showed that DEGs were mainly enriched in poly(A) RNA binding (Fig. 3c).

**Table 2:** Summary of the function of 10 hub genes

Symbol	Full name	Function
ITGAL	Integrin Subunit Alpha L	Involved in a variety of immune phenomena including leukocyte-endothelial cell interaction, cytotoxic T-cell mediated killing, and antibody dependent killing by granulocytes and monocytes. Contributes to natural killer cell cytotoxicity. Involved in leukocyte adhesion and transmigration of leukocytes including T-cells and neutrophils.contributes to apoptotic neutrophil phagocytosis by macrophages.
TLN1	Talin 1	Probably involved in connections of major cytoskeletal structures to the plasma membrane. High molecular weight cytoskeletal protein concentrated at regions of cell-substratum contact and, in lymphocytes, at cell-cell contacts
POLR2A	RNA Polymerase II Subunit A	DNA-dependent RNA polymerase catalyzes the transcription of DNA into RNA using the four ribonucleoside triphosphates as substrates. Largest and catalytic component of RNA polymerase II which synthesizes mRNA precursors and many functional non-coding RNAs. Forms the polymerase active center together with the second largest subunit. Pol II is the central component of the basal RNA polymerase II transcription machinery.
CD69	CD69 Molecule	Involved in lymphocyte proliferation and functions as a signal transmitting receptor in lymphocytes, natural killer (NK) cells, and platelets. By antigens, mitogens or activators of PKC on the surface of T and B-lymphocytes. By interaction of IL-2 with the p75 IL-2R on the surface of NK cells.
GZMA	Granzyme A	activates caspase-independent cell death with morphological features of apoptosis when delivered into the target cell through the immunological synapse. It cleaves after Lys or Arg. Cleaves APEX1 after ‘Lys-31’ and destroys its oxidative repair activity. Cleaves the nucleosome assembly protein SET after ‘Lys-189’, which disrupts its nucleosome assembly activity and allows the SET complex to translocate into the nucleus to nick and degrade the DNA.
VASP	Vasodilator Stimulated Phosphoprotein	Ena/VASP proteins are actin-associated proteins involved in a range of processes dependent on cytoskeleton remodeling and cell polarity such as axon guidance, lamellipodial and filopodial dynamics, platelet activation and cell migration. VASP promotes actin filament elongation. Plays a role in actin-based mobility of *Listeria monocytogenes* in host cells. Regulates actin dynamics in platelets and plays an important role in regulating platelet aggregation.
HNRNPUL1	Heterogeneous Nuclear Ribonucleoprotein U Like 1	Acts as a basic transcriptional regulator. Represses basic transcription driven by several virus and cellular promoters. When associated with BRD7, activates transcription of glucocorticoid-responsive promoter in the absence of ligand-stimulation. Plays also a role in mRNA processing and transport. Binds avidly to poly(G) and poly(C) RNA homopolymers in vitro.
SF1	Splicing Factor 1	Necessary for the ATP-dependent first step of spliceosome assembly. Binds to the intron branch point sequence (BPS) 5′-UACUAAC-3′ of the pre-mRNA. May act as transcription repressor.
SRRM2	Serine/Arginine Repetitive Matrix 2	Required for pre-mRNA splicing as component of the spliceosome.
ITGAV	Integrin Subunit Alpha V	The alpha-V (ITGAV) integrins are receptors for vitronectin, cytotactin, fibronectin, fibrinogen, laminin, matrix metalloproteinase-2, osteopontin, osteomodulin, prothrombin, thrombospondin and vWF. They recognize the sequence R-G-D in a wide array of ligands.

### Analysis of TF-target regulating networks

**Fig. 4:**
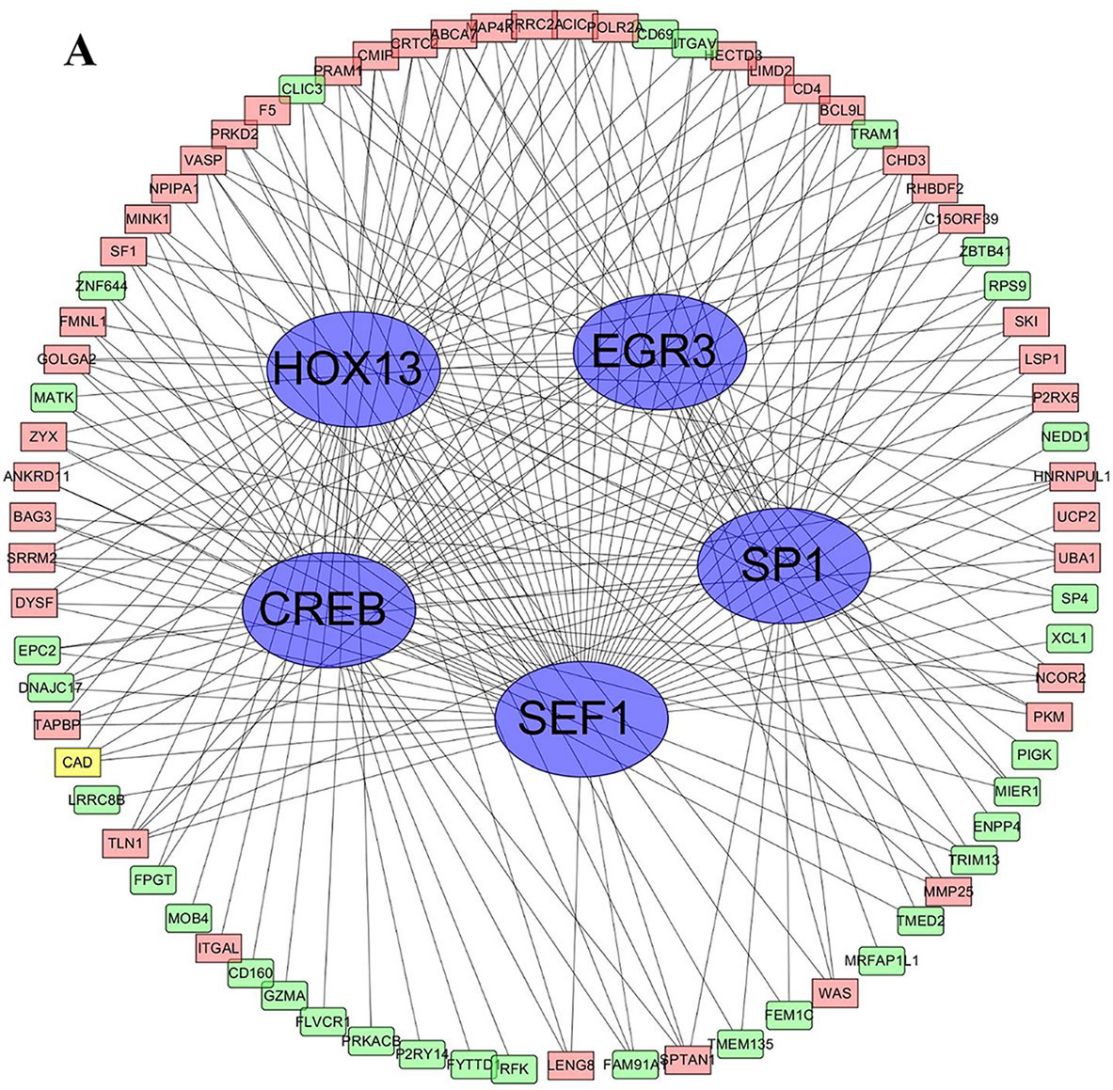
The TF-target regulating network including 214 DEGs and 5 transcription factors. Up-regulated genes are marked with light red; down-regulated genes are marked with light green, transcription factors are marked with blue

In the TF-target regulating network, 219 nodes were detected, including 214 DEGs and 5 transcription factors (TFs) (SP1,EGR3,CREB,SEF1,HOX13) (Fig. 4). Obviously, it is creditable to recognize that the TFs play a main regulatory role in network. Almost these 5 proteins encoded is zinc finger transcription factors that binds to several kinds of motifs of many promoters. We speculate that the predicted transcription factors may affect the process of early-onset atherosclerosis in familial hypercholesterolemia by activating or inhibiting transcription of these related differentially expressed genes.

## Discussion

FH is the most common genetic reason of cardiovascular disease which leads to premature atherosclerotic cardiovascular disease because of lifelong exposure to ascending low-density lipoprotein cholesterol (LDL-C) levels [23]. This genetic disorder influences the metabolism of low-density lipoprotein cholesterol (LDL-C), reducing the liver clearance of cholesterol-loaded LDL particles in the blood [24]. LDLR binds its low-density lipoprotein (LDL) particles to the plasma membrane, internalizes it, and then releases it in the low pH environment to degrade ribosomes, and enables cholesterol to occupy the microsomal enzyme 3-hydroxy-3- Methylglutaryl coenzyme A (HMG CoA) reductase, which is the rate-limiting step in cholesterol synthesis [25]. Plenty of experimental and epidemiological researches have proven the causal relationship of low-density lipoproteins (LDL) in the evolution of atherosclerosis and in the incidence of atherothrombotic complications like coronary heart disease (CHD) [26]. Even though the awareness of FH is increasing, this potentially fatal, treatable condition still remains underdiagnosed and undertreaded. Some traditional CVD risk factors ubiquitously exist in FH patients and already have been independently associated with CVD danger in the FH population. It is reported that some genetic factors such as single nucleotide polymorphisms (SNPs) and genetic variants like telomere length alteration in somatic cells, have been proven to predict the FH phenotype and CVD prognosis. Besides, certain circulating molecules, which play different roles in regulating the process of atherosclerosis, have been described as surrogate markers of CVD risk in FH populations. Hence understanding the changes in FH gene expression is of critical importance towards understanding the mechanism of disease progression and predicting the diagnostic or therapeutic targets of FH.

Bioinformatic technology has been generally applied to search for genes and molecules connected with the occurrence and development of relevant diseases and is regarded to be a promising technology for seeking targeted treatments. We can use the technology to find disease-related data from large open-sourced databases for analysis and identify the genes that are most concerned to the diseases. In present study, 102 mutual DEGs were identified in FH samples compared with healthy samples with 49 upregulated genes and 53 downregulated genes. By analyzing the PPI network, the 10 hub DEGs ITGAL, TLN1, POLR2A, CD69, GZMA, VASP, HNRNPUL1, SF1, SRRM2 and ITGAV were selected, among which ITGAL, TLN1, POLR2A, VASP, HNRNPUL1, SF1 and SRRM2 were expressed at higher levels while the expression levels of CD69, GZMA and ITGAV were lower in FH patients. The KEGG pathway analysis revealed that DEGs were significantly enriched in focal adhesion and glucagon signal pathway. Cell adhesion genes (ITGAL, TLN1), Poly(A) RNA binding-related genes (HNRNPUL1, SRRM2, SF1) and protein homotetramerization genes were enriched in these pathways. These significant DEGs and their functions were theorized to contribute to atherosclerosis development in FH patients.

The main risk of FH patients is the early onset of atherosclerosis and cardiovascular disease. Atherosclerosis is characterized by blood vessel wall hyperplasia, lipid accumulation in blood vessel wall, cytokine-activated macrophage invasion of blood vessel wall and formation of macrophage foam cells [27]. ITGAL belongs to the integrin α chain family, which encodes the integrin αL chain, and plays a role in T cell activation mainly through the contact of T cell receptors with antigens that bind to MHC molecules on antigen presenting cells [28]. Although no studies have reported that ITGAL is directly related to the development of FH or atherosclerosis. Previous studies have demonstrated that Ac-LDL uptake and TNF-α-dependent increase in THP-1 cells and the expression of OLR1, NOX2, NCF1, ITGA4 and ITGAL, suggest that ITGAL regulates Ac-LDL uptake and affects the formation of foam cells in macrophages [29]. We observed that GO annotations related to this gene include protein heterodimer activity and cell adhesion molecule binding, which may be related to abnormal immune cell adhesion or accumulation of metabolites in blood vessels. In addition, there are reports that using ItgaL−/−null NOD/LtJ mice, genetic defects of ItgaL can prevent the occurrence of hyperglycemia. Animal experiments have shown that lack of ItgaL can prevent insulin resistance, while lack of Itgb2 can provide protection. Transferring splenocytes lacking ItgaL to NOD/Rag-1 experimental mice does not lead to the development of diabetes, which suggests that ItgaL has a role in NOD/LtJ T cell activation [30]. John H Chidlow Jr. et al. used an external parallel plate flow chamber model and it was found that gene deletion of ItgaM completely prevented neutrophils from agglutinating into the endothelium stimulated by VEGF-A, while lack of ItgaL only weakened the adhesion of neutrophils. The lack of ItgaM does significantly reduce the rolling of neutrophils, but the lack of ItgaL does not. They also found that genetic defects in ItgaL or ItgaM do significantly inactivate T cell adhesion to VEGF-A-stimulated colonic endothelium [31]. This means that the ITGAL gene we identified may be related to the abnormal deposition of atherosclerotic endothelium.

TLN1 are reported to be associated with important biological processes, including platelet degranulation, muscle contraction, cytoskeletal anchoring at plasma membrane, cell-cell junction assembly and cell-substrate junction assembly [32]. This gene probably involved in connections of major cytoskeletal structures to the plasma membrane. High molecular weight cytoskeletal protein concentrated at regions of cell-substratum contact and, in lymphocytes, at cell-cell contacts. Diseases associated with TLN1 include Leukocyte adhesion deficiency, type I and Leukocyte adhesion deficiency, type Iii. As we all known, the universally expressed cytoskeletal protein talin (Tln) is a constituent of muscle costameres that connects integrins ultimately with the sarcomere. And there are two talin genes, Tln1 and Tln2 expression where Tln2 is the dominant isoform [33]. A study tested the function of both two Tln forms in myocardium in postnatal CMs. Recent studies in non-muscle cells have also found that Tln is a key regulator of force transmission and transduction. This is a particularly important feature of the heart muscle. The myocardium is an organ that is continuously subjected to mechanical force under basic conditions and must adapt to mechanical changes under physiological pressure or pathological conditions [34, 35]. Interestingly, researchers found that global deletion of Tln2 in mice had no structural or functional variations in heart, perhaps on account of up-regulated CM Tln1 [32]. The results revealed that CM Tln2 was indispensable for appropriate β1D-integrin expression and that presumably Tln1 could take the place of Tln2 in preserving heart function, however, that lack of both Tln forms from the heart-muscle cell resulted in myocyte instability and a dilated cardiomyopathy.

In addition, our present analysis as well allowed the identification of some TFs (SP1,EGR3,CREB,SEF1,HOX13) associated with FH, which suggests that these genes play important roles in FH. Based on the current literature, we discuss below the association between FH and the transcription factors identified herein. The protein encoded by SP1 is also a zinc finger transcription factor that combines with GC-rich motifs of many promoters. Besides SP1 can activate or repress transcription in answer to physiological and pathological stimuli. It binds with high affinity to GC-rich motifs and regulates the expression of numerous genes involved in various processes such as cell growth, apoptosis, differentiation and immune responses [36]. We speculate that the expression level of sp1 may regulate the calcification of collagen in atherosclerotic plaques. Interestingly, it has been demonstrated that unstable (known as noncalcified) plaques undergo thinning of the fibrous cap prior to rupture, possibly as a result of macrophages releasing proteolytic matrix-degrading enzymes which may degrade the fibrous cap3 It’s reported SP1 was highly regulated by post-translational modifications (phosphorylations, sumoylation, proteolytic cleavage, glycosylation and acetylation) and also bond the PDGFR-alpha G-box promoter [37]. Besides, this transcription factor may have a role in modulating the cellular response to DNA damage. According to the latest reports, it was found that because of the descending recruitment of SP1 to SCARB1 promoter the SCARB1 was downregulated by DNMT3b. In our view, this discovery will provide novel insight into an underlying mechanism for atherosclerosis of FH [38]. Another transcription factor EGR3, Early Growth Response 3, remains with the EGR family of C2H2-type zinc-finger proteins. It is reported EGR3 was an immediate-early growth response gene which was induced by mitogenic stimulation and it functioned in a wide variety of processes including muscle development, lymphocyte development, endothelial cell growth and migration, and neuronal development. Previously reported that diseases associated with EGR3 include bipolar I disorder and chondromalacia of patella. Based on research fruits, among its related pathways are Circadian rhythm related genes and Calcineurin-regulated NFAT-dependent transcription in lymphocytes. Jun-ichi Suehiro et al. displayed that in HUVECs, Egr-3 showed more pronounced, delayed, and sustained induction in contrast with Egr-1. Furthermore, deletion of Egr-3 remarkably vitiated the proliferation, migration, and tube formation of endothelial cells and hindered monocyte adhesion mediated by VEGF. From the above, these findings suggest that Egr-3 plays a critical role of VEGF signaling in activated endotheliocytes. So EGR3 is likely to be a potential therapeutic target for a preventive against vasculopathic diseases. CREB gene encodes a transcription factor that is a member of the leucine zipper family of DNA binding proteins. This protein binds as a homodimer to the cAMP-responsive element, an octameric palindrome. The protein is phosphorylated by several protein kinases, and induces transcription of genes in response to hormonal stimulation of the cAMP pathway. It is a phosphorylation-dependent transcription factor, which is Involved in different cellular processes including the synchronization of circadian rhythmicity and the differentiation of adipose cells. The suppressor of essential function 1 (SEF1) is a zinc finger transcription factor and this fungal transcription factor regulates genetic middle homology region. Some studies reported that Sef1 reacted to deficient Fe-S cluster synthesis via regulated changes in its subcellular location; it was maintained in the nucleus resulting in the revulsive expression of the iron regulon [39]. The homeobox transcription factor Hox13 is a member of the Hox family containing homeobox genes and encodes DNA binding proteins. In previous studies, the structure, genomic organization, expression patterns and biological functions of the Hox family are highly conserved [40]. In vertebrates, axial Hox expression was observed in neural tubes and some paraxial mesoderm derivatives, while in arthropods, in the ventral nerve cord, visceral mesoderm and Hox gene expression was found in the epidermis [41]. Albeit no reports were seen about HOX13 has a regulatory relationship with FH or atherosclerosis, we believe that it is necessary to further study the underlying mechanism of hox13.

Even though the rigorous bioinformatic analysis was carried out in present study, there are still some weaknesses. The quantity of data in this study is limited so that some deviations may exist in the results. Enlarging the samples can enhance the accuracy of the analysis findings. Beyond that, despite it can be explained to some degree that the hub molecules and TFs are closely associated with the development of FH and may also function as potential markers for therapeutic targets, specific mechanism researched are still of great necessary on animal or cell experiments.

## Conclusion

Above all, findings in the current study demonstrated that the development of atherosclerosis might be the result of imbalance between macrophages and fibrosis. Specifically, up-regulated ITGAL, TLN1, POLR2A, VASP, HNRNPUL1, SF1, SRRM2, and down-regulated, CD69, GZMA,ITGAV performed important promotional effects for the formation of atherosclerotic plaques those with FH. Moreover, (SP1, EGR3, CREB, SEF1, HOX13) were the potential transcription factors for DEGs and could serve as underlying targets for AS rupture prevention. These findings provide a theoretical basis for us to understand the potential etiology of the occurrence and development of AS in FH patients and we may be able to find potential diagnostic and therapeutic targets.

## Data Availability

The gene expression profiles of GSE13985 and GSE6054 were downloaded from Gene Expression Omnibus (GEO) (https://www.ncbi.nlm.nih.gov/geo/query/acc.cgi?acc=GSE13985 and https://www.ncbi.nlm.nih.gov/geo/query/acc.cgi?acc=GSE6054).

## Abbreviations

FH: Familial hypercholesterolemia
LDL-C: Low-density lipoprotein cholesterol
AS: Atherosclerosis
CVD: Cardiovascular disease
DEGs: Differently expressed genes
GEO: Gene expression omnibus
KEGG: Kyoto encyclopedia of genes and genomes
GO: Gene ontology
STRING: Search tool for the retrieval of interacting genes
PPIs: Protein-protein interactions
ITGAL: Integrin subunit alpha L;TLN1:Talin
POLR2A: RNA polymerase II subunit A
CD69: CD69 molecule
GZMA: Granzyme A
VASP: Vasodilator stimulated phosphoprotein
HNRNPUL1: Heterogeneous nuclear ribonucleoprotein U like 1
SF1: Splicing factor 1
SRRM2: Serine/arginine repetitive matrix 2
ITGAV: Integrin subunit alpha V
EGR3: Early growth response 3
CREB: cAMP responsive element binding protein
SEF1: Suppressor of essential function 1
HOX13: Homeobox transcription factor 13

## References

[1] Bouhairie VE, Goldberg AC (2015). Familial hypercholesterolemia. Cardiol Clin.

[2] Goldberg AC, Hopkins PN, Toth PP (2011). Familial hypercholesterolemia: screening, diagnosis and management of pediatric and adult patients: clinical guidance from the National Lipid Association Expert Panel on Familial Hypercholesterolemia. J Clin Lipidol.

[3] Raal FJ, Santos RD (2012). Homozygous familial hypercholesterolemia: current perspectives on diagnosis and treatment. Atherosclerosis.

[4] Singh S, Bittner V (2015). Familial hypercholesterolemia--epidemiology, diagnosis, and screening. Curr Atheroscler Rep.

[5] Shi Z, Yuan B, Zhao D, Taylor AW, Lin J, Watts GF (2014). Familial hypercholesterolemia in China: prevalence and evidence of underdetection and undertreatment in a community population. Int J Cardiol.

[6] Wang X, Jiang L, Sun LY (2018). Genetically confirmed familial hypercholesterolemia in outpatients with hypercholesterolemia. J Geriatr Cardiol.

[7] Goldstein JL, Brown MS (2009). History of discovery: the LDL receptor. Arterioscler Thromb Vasc Biol.

[8] Benito-Vicente A, Uribe KB, Jebari S, Galicia-Garcia U, Ostolaza H, Martin C (2018). Familial hypercholesterolemia: the most frequent cholesterol metabolism disorder caused disease. Int J Mol Sci.

[9] Santos PC, Morgan AC, Jannes CE (2014). Presence and type of low density lipoprotein receptor (LDLR) mutation influences the lipid profile and response to lipid-lowering therapy in Brazilian patients with heterozygous familial hypercholesterolemia. Atherosclerosis.

[10] Hovingh GK, Davidson MH, Kastelein JJ, O'Connor AM (2013). Diagnosis and treatment of familial hypercholesterolaemia. Eur Heart J.

[11] Omer L, Hudson EA, Zheng S, Hoying JB, Shan Y, Boyd NL (2017). CRISPR correction of a homozygous low-density lipoprotein receptor mutation in familial hypercholesterolemia induced pluripotent stem cells. Hepatol Commun.

[12] Mao C, Howard TD, Sullivan D (2017). Bioinformatic analysis of coronary disease associated SNPs and genes to identify proteins potentially involved in the pathogenesis of atherosclerosis. J Proteom Genom Res.

[13] Lei F, Zhang H, Xie X (2019). Comprehensive analysis of an lncRNA-miRNA-mRNA competing endogenous RNA network in pulpitis. PeerJ.

[14] Zhao Q, Sun D, Li Y, Qin J, Yan J (2019). Integrated analyses of lncRNAs microarray profiles and mRNA-lncRNA coexpression in smooth muscle cells under hypoxic and normoxic conditions. Biosci Rep.

[15] Chen DQ, Kong XS, Shen XB (2019). Identification of differentially expressed genes and signaling pathways in acute myocardial infarction based on integrated bioinformatics analysis. Cardiovasc Ther.

[16] Vernon ST, Hansen T, Kott KA, Yang JY, O'Sullivan JF, Figtree GA (2019). Utilizing state-of-the-art “omics” technology and bioinformatics to identify new biological mechanisms and biomarkers for coronary artery disease. Microcirculation.

[17] Yin Y, Zou YF, Xiao Y, et al. Identification of potential hub genes of atherosclerosis through bioinformatic analysis. J Comput Biol. 2020. 10.1089/cmb.2019.0334. 10.1089/cmb.2019.0334 32286084

[18] Wang H, Liu D, Zhang H (2019). Investigation of the underlying genes and mechanism of macrophage-enriched ruptured atherosclerotic plaques using bioinformatics method. J Atheroscler Thromb.

[19] Wang HX, Zhao YX (2016). Prediction of genetic risk factors of atherosclerosis using various bioinformatic tools. Genet Mol Res.

[20] Zhang N, Chen Y, Lou S, Shen Y, Deng J (2019). A six-gene-based prognostic model predicts complete remission and overall survival in childhood acute myeloid leukemia. Onco Targets Ther.

[21] Li G, Wu X-J, Kong X-Q, Wang L (2015). Cytochrome c oxidase subunit VIIb as a potential target in familial hypercholesterolemia by bioinformatical analysis. Eur Rev Med Pharmacol Sci.

[22] Tang Q, Wang Q, Zhang Q (2017). Gene expression, regulation of DEN and HBx induced HCC mice models and comparisons of tumor, para-tumor and normal tissues. BMC Cancer.

[23] Marks D, Thorogood M, Neil HAW, Humphries SE (2003). A review on the diagnosis, natural history, and treatment of familial hypercholesterolaemia. Atherosclerosis.

[24] Brandts J, Dharmayat KI, Ray KK, Vallejo-Vaz AJ (2020). Familial hypercholesterolemia: is it time to separate monogenic from polygenic familial hypercholesterolemia?. Curr Opin Lipidol.

[25] Kassim SH, Vandenberghe LH, Hovhannisyan R, Wilson JM, Rader DJ (2011). Identification and functional characterization in vivo of a novel splice variant of LDLR in rhesus macaques. Physiol Genomics.

[26] Xu S, Weng J (2020). Familial hypercholesterolemia and atherosclerosis: animal models and therapeutic advances. Trends Endocrinol Metab.

[27] Falk E (2006). Pathogenesis of atherosclerosis. J Am Coll Cardiol.

[28] Blanco FJ, Ojeda-Fernandez L, Aristorena M (2015). Genome-wide transcriptional and functional analysis of endoglin isoforms in the human promonocytic cell line U937. J Cell Physiol.

[29] Yamagata K, Tusruta C, Ohtuski A, Tagami M (2014). Docosahexaenoic acid decreases TNF-alpha-induced lectin-like oxidized low-density lipoprotein receptor-1 expression in THP-1 cells. Prostaglandins Leukot Essent Fatty Acids.

[30] Glawe JD, Patrick DR, Huang M, Sharp CD, Barlow SC, Kevil CG (2009). Genetic deficiency of Itgb2 or ItgaL prevents autoimmune diabetes through distinctly different mechanisms in NOD/LtJ mice. Diabetes.

[31] Chidlow JH, Glawe JD, Alexander JS, Kevil CG (2010). VEGF164 differentially regulates neutrophil and T cell adhesion through ItgaL- and ItgaM-dependent mechanisms. Am J Physiol Gastrointest Liver Physiol.

[32] Manso AM, Okada H, Sakamoto FM (2017). Loss of mouse cardiomyocyte talin-1 and talin-2 leads to beta-1 integrin reduction, costameric instability, and dilated cardiomyopathy. Proc Natl Acad Sci U S A.

[33] Manso AM, Li R, Monkley SJ (2013). Talin1 has unique expression versus talin 2 in the heart and modifies the hypertrophic response to pressure overload. J Biol Chem.

[34] Austen K, Ringer P, Mehlich A (2015). Extracellular rigidity sensing by talin isoform-specific mechanical linkages. Nat Cell Biol.

[35] Elosegui-Artola A, Oria R, Chen Y (2016). Mechanical regulation of a molecular clutch defines force transmission and transduction in response to matrix rigidity. Nat Cell Biol.

[36] O’Connor L, Gilmour J, Bonifer C (2016). The role of the ubiquitously expressed transcription factor Sp1 in tissue-specific transcriptional regulation and in disease. Yale J Biol Med.

[37] Koivisto U-M, Palvimo JJ, Jannet OA, Kontula K (1994). A single-base substitution in the proximal Spl site of the human low density lipoprotein receptor promoter as a cause of heterozygous familial hypercholesterolemia. Genetics.

[38] Guo W, Zhang H, Yang A (2020). Homocysteine accelerates atherosclerosis by inhibiting scavenger receptor class B member1 via DNMT3b/SP1 pathway. J Mol Cell Cardiol.

[39] Ror S, Panwar SL (2019). Sef1-regulated iron regulon responds to mitochondria-dependent iron-sulfur cluster biosynthesis in Candida albicans. Front Microbiol.

[40] Ros MA (2016). HOX13 proteins: the molecular switcher in Hoxd bimodal regulation. Genes Dev.

[41] Sheth R, Barozzi I, Langlais D (2016). Distal limb patterning requires modulation of cis-regulatory activities by HOX13. Cell Rep.

